# Can government subsidies promote the construction of “Blue Basic Farmland”? Analysis of multi-subject management model in mariculture areas

**DOI:** 10.1016/j.heliyon.2024.e27059

**Published:** 2024-02-24

**Authors:** Zicheng Wang, Shan Zheng

**Affiliations:** a Facultad de Comercio y Turismo, Universidad Complutense de Madrid, Madrid, 2 28040, Spain; b Ocean University of China, Qingdao, 266100, PR China

**Keywords:** Blue basic farmland, Fishery subsidies, Fishermen, Evolutionary game

## Abstract

The implementation of the “Blue Basic Farmland” system for farming ocean is a crucial strategy to ensure food security. This study simulates and analyzes the impact of government fishery subsidies on promoting “Blue Basic Farmland” construction. A tripartite evolutionary game model involving the government, fishermen and fishery enterprises was used”. Subsequently, the fishery subsidy strategy of “Blue Basic Farmland” construction was simulated and analyzed. The following findings are derived. First, fishery subsidies contributed to “Blue Basic Farmland” construction by promoting the participation of fishing enterprises and supporting fishermen. Second, the amounts of fishery subsidies were not the high the better, and different amounts of fishery subsidies had varying effects. Third, subsidizing fishery enterprises was more effective in promoting the construction of “Blue Basic Farmland” compared to subsidizing fishermen. In light of these findings, we proposed the following policy recommendations. The government ought to establish subsidies specifically tailored for “Blue Basic Farmland” construction, while also judiciously controlling the subsidies amounts. Moreover, the focus of these subsidies should be directed towards supporting fishery enterprises.

## 1 Introduction

The aquaculture areas of the ocean are an important base for marine food production, and the main products are algae and animal food [1]. Compared to terrestrial production models that grow feed grains for meat production, mariculture has higher productivity and nutrient conversion efficiency [2]. By 2050, marine edible food could increase by 21–44 million tons, an increase of 36–74% compared to current production [3]. In recent years, the continued decline in arable land per capita has posed a serious threat to food security. Although it is feasible to secure food supply by expanding the size of arable land, it may destroy biodiversity and contributes to global climate change, and even threatens the normal functioning of other ecosystems [4]. Given this background, designating permanent basic farmland is a crucial step in stabilizing food supply and guarantee national food security [5]. It can protect high-quality farmland around cities that is easily occupied, and thus strictly control the use of farmland for accelerated urban expansion [6]. Similarly, under the current situation of land resources tension and ecological environment deterioration, marine fishery resources continue to decline, the protection of aquaculture waters must be strengthened to ensure and expand the production capacity of mariculture, so as to alleviate the pressure of food security brought about by the growth of animal food consumption of the population at this stage.

In China, basic farmland refers to the arable land that cannot be occupied based on the requirement for agricultural produce by the people and economic development, as determined by the overall land use plan. On this basis, professor Han further proposed the idea of building a “Blue Basic Farmland” system [7]. The “Blue Basic Farmland” (BBF) is the farming ocean area that cannot be changed according to the ocean use plan. Unlike the basic farmland on land, the Blue Basic Farmland establishes that there is competition between mariculture and fishermen for the use of the ocean [8]. In the case of Blue Basic Farmland, the fishermen need to withdraw from the fishing industry and the fishery enterprises to participate in the operation. Therefore, mobilize the enthusiasm of fishery enterprises to operate and make up for the loss of fishermen to become the key to the BBF construction. It should not be overlooked that the farming ocean area is geographically and spatially public, and it is impossible to achieve harmony through market regulation, thus creating the need and dependence on external policies and requiring government intervention. Fishery subsidies are a common government measure aimed at supporting the industry. Recognizing the significance of these subsidies, what kind of subsidies strategy should the government adopt to promote the construction of Blue Basic Farmland?To summarize, we construct a multi-subject model for BBF construction, analyze whether it should be subsidized or not, and examine the subsidies conditions in BBF construction. Further, we simulate and analyze the influence of fishery subsidies amount and subsidies recipients on the game system's evolution of BBF construction based on survey data. The findings of this study can be used to inform policymakers and stakeholders in the development of sustainable aquaculture practices and the allocation of subsidies in the fishery sector. The “Blue Basic Farmland” system can serve as a model for other countries and regions looking to promote sustainable aquaculture and protect their marine ecosystems.

There are two innovations in this paper. First, we propose and analyze a multi-subject model of BBF. Unlike the construction of basic farmland on land, the leader of BBF is government, the operator is fishery enterprise, and fishermen need to quit fishing. Second, the BBF system has not yet been implemented. We simulate the evolution of fishery subsidies policy using evolutionary game and numerical simulation techniques, test the policy effect through simulation experiments, and provide a reference for countries to further improve the management system for the use of maritime areas.

## 2 Literature review

### 2.1 Decline of terrestrial farmland and protection of basic farmland

Basic farmland system establishment was due to the gradual decrease in arable land and the increasing trend of non-farming of arable land, which may affect national food security [5]. For one thing, as the population grows and the social economy develops, the arable land is decreasing. In the United States, arable land has decreased while grassland and forest land have slightly increased [9]. In the Czech Republic, larch and spruce are planted on agricultural land to convert it into woodland [10]. In Poland, urban expansion and the rapid urbanization of rural regions have led to the conversion of agricultural land to other purposes [11]. In contrast to abandoned arable land, woodland and grassland, agricultural land in the rocky mountain areas of northern China has experienced strong soil erosion [12]. Furthermore, non-farming has led to a structural undersupply of farmland. Farmland utilization is influenced by biophysical, bioclimatic and agro-socio-economic factors [13]. Yang and Zhang found that the “one village, one product” strategy posed a threat to food security in 47 counties of China, with a 29.53% increase in the cropland stress index [14].

As arable land gradually diminishes, the government started to propose farmland protection measures in order to guarantee national food security. Bański and Kamińska diagnosed and identified the trends in the structure of farmland use in Central European countries and spatial differentiation this structure exhibits. The results show that the national agricultural policy and the agricultural policy of the European Union seem to have an increasing influence on the crop structure of arable land [15]. In the case of territorial spatial planning, the territorial planning documents that have been approved reflect the state's policy for a particular area, as well as represent a compromise between the state, its representatives, property owners, and the community and its individual members [16]. Using an automatic model of land system cells, Wang et al. optimized China's urban and agricultural land spatial distribution, and the results of the study showed that by optimizing the spatial distribution of agricultural land, it is possible to reduce the fragmentation of arable land and increase the connectivity of ecological patches. This can lead to a more efficient use of agricultural resources, while also promoting ecological conservation and biodiversity [17].

In terms of laws and regulations, Kowalczyk et al. found that national laws influence agricultural land markets and farm structures [18]. The permanent basic farmland designation has been successful in preserving agricultural land in China, which is crucial for the country's food security. It has also helped to reduce the loss of farmland to urbanization and industrialization, which can be detrimental to the environment and rural communities [19]. Chen et al. proposed a model for the delineation of permanent basic farmland, integrating principles such as spatial continuity, soil quality, and land use efficiency. This model serves to identify and prioritize areas for the permanent protection and utilization of farmland [6]. Qian et al. introduced the land evaluation and site assessment system to China, which is designed to provide a comprehensive evaluation of the suitability of land for various uses, such as agriculture, forestry, and urban development. It takes various factors into account such as soil quality, topography, climate, and water resources to determine the most appropriate use for the land [20]. Based on this system, Chen et al. proposed an extensive quality assessment index system for farmland and established a technical framework based on “sequential priority, quality screening, and scale constraint” [5]. In terms of farmland protection policies, Canada has implemented best management practice incentives to protect agricultural land in Ontario [21]. Following the implementation of the arable land protection policy, the ecological governance and conservation level, agricultural production motivation, and farmer satisfaction generally fall within the moderate to low range, with only the quantity of arable land protection reaching a medium to high level [22]. In terms of farmland conservation from farmers' perspective, Abera et al. studied the factors affecting smallholder land management practices. The study found that farmers' decisions on land management practices were influenced by a range of factors, including access to credit, land tenure security, market opportunities, and agro-ecological conditions [23]. Due to the existence of collective action dilemma, Zhang et al. examined the compound causal mechanism of antecedent conditions, including perceived benefits and policy incentives, on the willingness to cooperate in farmland conservation using questionnaire data obtained from 197 farmers in China [24].

There are of course shortcomings in farmland protection measures. Vietnam's farmland protection policy has improved the economic efficiency of rice land, but may lead to a misallocation of resources in favor of the rich while making the poor worse off [25]. As policies for farmland protection, regulatory and payment instruments operate independently or complement each other. However, under China's policy of total farmland control and balance of farmland occupation, alternative relationships exist among regulatory instruments and payment instruments [26]. The Chinese system of balancing farmland occupation and compensation has led to the transformation of high-quality farmland into industrial and residential land, while simultaneously replenishing low-quality farmland. This approach prioritizes the protection of farmland quantity over farmland quality [19]. Therefore, from the perspective of land and ocean integration, establishing BBF to alleviate the problem of decreasing land farmland quantity provides a new idea to protect basic farmland and guarantee national food security.

### 2.2 Land and ocean integration and blue basic farmland construction

The ocean is a new space to guarantee national food security [27]. Over 3 billion people relying on fish and other seafood as their primary source of protein [28]. In the context of exhaustion of land development potential, it is necessary to build a marine food supply system based on the integrated perspective of land and ocean, with marine space as the basis, marine biological resources development and utilization as the means, and marine aquatic products production and its associated industries as the carrier [29]. Marine products offer a promising solution to address the challenge of global food security. However, sustainable management practices are vital to ensure the long-term viability of marine ecosystems and the communities that rely on them [30]. Ahmed and Lorica found that aquaculture can improve household food security and nutritional status by increasing income levels, marginal productivity of agricultural labor, and consumption levels in three ways [31]. Some studies suggest that fisheries and aquaculture can make a significant difference in alleviating poverty and improving food security, while others argue that these industries can have negative impacts on the environment and local communities [32]. In developing countries such as Asia and Africa, where seafood is an important source of food, Olaifa et al. noted that fish is one of the few sources of animal protein available to many Nigerians, and that a one percentage point increase in total fish production would contribute to a 655.88% increase in food security [33]. In sub-Saharan Africa, the demand for fish exceeds the supply due to a shift in diet towards fish, economic and population growth trends, thus posing a threat to food security [34]. March and Failler showed that small-scale aquaculture and diversification of inland fisheries can significantly impact the livelihoods, food security and climate resilience of inland communities [35]. Belton et al. assessed the changing patterns of fish consumption and its impact on food and nutrition security in Bangladesh. The study found that fish consumption has increased over the past decades, with a greater proportion of fish being sourced from mariculture. However, this increase in fish consumption has not necessarily translated into improved nutrition outcomes for all populations, particularly those who are socio-economically disadvantaged or geographically remote [36].

The supply of marine food is dependent on the mariculture industry. Based on future fish supply and demand projections, Solomon Islands needs to invest in mariculture to ensure food security [37]. Due to the competition for industrial use of the ocean, it is particularly vital to designate ocean areas for aquaculture. Currently, there is no clear international concept of BBF, only functional areas for mariculture. There is still a large area of deep ocean to develop mariculture [38], but the BBF has not yet been developed due to the strong technology, large capital investment and high risk.

The BBF construction cannot be achieved without the support of government subsidies. Government subsidies creates a two-fold motivational effect from both the producer and consumer side [39]. The Chinese government uses subsidies for fuel, pelagic fisheries, infrastructure, and various “green boxes” to increase fish production [40]. To supporting the development of the fisheries sector, improving the competitiveness of Chinese fishing vessels, and ensuring food security, the Chinese central government provided RMB 40.383 billion in fishery subsidies in 2013, among them, fuel subsidies accounted for 94% [41]. However, these subsidies have also been criticized for contributing to overfishing and the depletion of fish stocks in Chinese waters, as well as for causing environmental damage and increasing greenhouse gas emissions due to the use of fossil fuels. Recently, the Chinese government has taken steps to reform its fishery subsidies, including reducing fuel subsidies and promoting sustainable fishing practices. Guillen et al. provided insights into the importance of mariculture subsidies by comparing the subsidies evolution for mariculture growth in different EU countries from 2000 to 2020. The study argue that subsidies are essential to ensure the continued growth and sustainability of mariculture in the EU. However, they also acknowledge that subsidies can create distortions in the market and that there is a need for more transparency and accountability in their allocation and use [42]. Peñalosa Martinell et al. analyzed the impact of fuel and energy consumption subsidies policies on mariculture production in Mexico and propose the application of subsidies for sustainable mariculture development [43]. Mallory studied the impact of subsidies inequity on fish stock decline and governance of small-scale marine fisheries. Findings show that fuel subsidies for small-scale fishers are unequally distributed and jeopardize fishers' livelihoods [44]. Formenti shed new light on the determinants of government transparency in fishery subsidies by screening the text of Chinese and U.S. notifications under the Subsidies and Countervailing Measures Agreement [45]. The research provides insight into the complex factors that influence government transparency in fishery subsidies and highlights the importance of continued monitoring and analysis of these policies.

### 2.3 Literature gaps

First, existing studies have focused on terrestrial agriculture to ensure food security, ignoring to some extent the BBF function of the ocean. In fact, seafood is an indispensable part of the human food delivery chain. At present, the development of terrestrial farmland has reached its maximum capacity, and mariculture has gradually evolved into a significant focus to ensure national food security. Therefore, constructing BBF is an urgent, important and novel research problem.

Second, there are more studies on terrestrial ‘basic farmland’ in the literature than on BBF. There is a clear difference between the BBF construction and land “basic farmland”. The construction of “basic farmland” on land can be completely led by the government, and the scope of fixed basic farmland can be defined based on arable land distribution. However, the BBF construction involves the livelihood of fishermen and the cultivation of mariculture business entities, which involves more interest subjects, so the construction mode led by a single government entity is not feasible.

## 3 Methodology

### 3.1 Research area

**Fig. 1:**
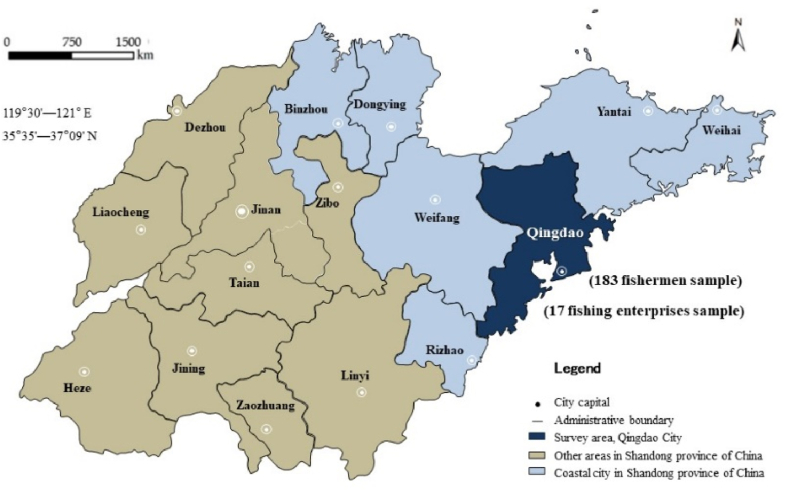
Map of the study area.

This study was conducted in Qingdao City, China (Fig. 1). In 2022, the survey conducted by the BBF construction group for Qingdao municipal government, fishermen and fishery enterprises. The sample involved 17 fishing enterprises and 183 fishermen. Due to the relatively limited number of fishery enterprises, this study adopts typical sample survey for sample selection. A list containing all fishery enterprises was first constructed and ranked according to enterprise size criteria, and the top 17 fishery enterprises were selected from the ranked list as the survey sample. In addition, in order to effectively represent the broad and diverse group of fishermen in Qingdao, we used a combination of stratified and simple random sampling.

First, based on geographic location and fishing methods, Jimo District, Huangdao District, Chengyang District and Jiaozhou District were selected as sample counties. Second, three sample villages were selected from each sample county based on the level of fishery development, fishery output value, distribution of fishery resources and other factors. In selecting the sample villages, consideration was given to ensure that all types of fishery activities were covered and that the diversity of regional fisheries was adequately reflected. Again, the fishermen in each sample village were stratified into high, medium, and low groups based on factors such as fishing scale, fishing experience, and fishing equipment. Such stratification can better reflect the differences in fishery practitioners at different levels. Next, from each sample group, six to seven fishermen were selected as a sample using random sampling. Such random sampling helps to ensure the representativeness of the sample and the validity of statistical inference. Ultimately, a total of 183 representative samples of fishermen were drawn through this sampling process to ensure adequate coverage of all strata, thus allowing for a more comprehensive and accurate understanding of the characteristics and circumstances of the fishermen groups. The parameters obtained from the survey, which were used to assign values in the numerical simulation model.

### 3.2 Evolutionary game method

**Fig. 2:**
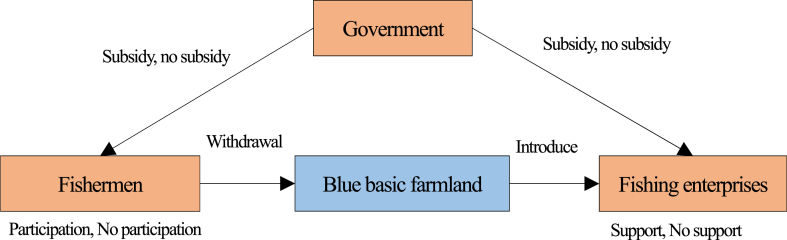
BBF multi-subject construction model.

The establishment of mariculture areas has resulted in a reduction of available ocean space for fishing, causing damage to the interests of fishermen and even affecting their livelihoods [8]. As a result, fishermen may either support or oppose the BBF construction. While the inshore BBF is mostly saturated, mariculture in the offshore BBF is more technically complex and risky. Fishery enterprises may choose to participate in the BBF construction with the motive of chasing profits, or they may not participate in the BBF construction to avoid risks. The government is the designer and implementer of the BBF policy. To facilitate the execution of the BBF policy, the government may provide subsidies to fishery enterprises, aiming to incentivize their engagement in BBF construction. Additionally, the government can also offer subsidies to fishermen, to withdraws their fishing licenses, and guide them to reduce their fishing boats and switch to other industries. This multi-subject model of BBFs involves the government, fishermen, and fishery enterprises, as illustrated in Fig. 2.

The probability of fishery enterprises participating in the BBF construction is x(0≤x≤1), and not participating in the BBF construction is 1−x. The costs and benefits associated with the involvement of fishery enterprises in BBF construction are denoted as $p_{x}$ and $r_{x}$, respectively. The costs and benefits incurred by fishery enterprises that refrain from participating in BBF construction are designated as $p_{1-x}$ and $r_{1-x}$, respectively. Fishermen can choose to support or not to support the BBF construction. The probabilities of fishermen supporting and not supporting the BBF construction are y(0≤y≤1), and 1−y. The cost for the fishermen to support the BBF construction is $p_{y}$, and fishermen's benefit is $r_{y}$. The fishermen's cost and benefit of not supporting the BBF construction is $p_{1-y}$ and $r_{1-y}$, respectively. Government-issued fishery subsidies can influence the strategic decisions made by both fishery enterprises and fishermen. The probability of the government providing and not providing fishery subsidies to BBF are z(0≤z≤1) and 1−z. If the government provides fishery subsidies, the cost it pays and the utility it obtains are $p_{z}$ and $r_{z}$, respectively. If the government does not provide fishing subsidies, the cost it pays and the utility it obtains are $p_{1-z}$ and $r_{1-z}$, respectively.

Assuming that the fishery subsidizes the BBF construction is m, the coefficients for subsidizing fishery enterprises and fishermen are $a_{1}$ and $a_{2}$, $a_{1}+a_{2}=1$. Table 1 displays the benefit matrix of the tripartite game of BBF construction, as determined by the aforementioned cost-benefit analysis.

**Table 1:** The game revenue matrix of BBF construction.

Fishery enterprises	Fishermen	Government subsidies (z)	No government subsidies (1-z)
Participation (x)	Support (y)	$r_{x}-p_{x}+a_{1}m$ $r_{y}-p_{y}+a_{2}m$ $r_{z}-p_{z}-m$	$r_{x}-p_{x}$ $r_{y}-p_{y}$ $r_{1-z}-p_{1-z}$
	Not supported (1 - y)	$r_{x}-p_{x}+a_{1}m$ $r_{1-y}-p_{1-y}$ $r_{z}-p_{z}-a_{1}m$	$r_{x}-p_{x}$ $r_{1-y}-p_{1-y}$ $r_{1-z}-p_{1-z}$
Non-participation (1-x)	Support (y)	$r_{1-x}-p_{1-x}$ $r_{y}-p_{y}+a_{2}m$ $r_{z}-p_{z}-a_{2}m$	$r_{1-x}-p_{1-x}$ $r_{y}-p_{y}$ $r_{1-z}-p_{1-z}$
	Not supported (1 - y)	$r_{1-x}-p_{1-x}$ $r_{1-y}-p_{1-y}$ $r_{z}-p_{z}$	$r_{1-x}-p_{1-x}$ $r_{1-y}-p_{1-y}$ $r_{1-z}-p_{1-z}$

#### 3.2.1 The construction of replication dynamic equation for all subjects

Referring to the evolutionary game benefit matrix of BBF construction in Table 1, we calculate the expected benefits for fishery enterprises participating in BBF construction, the expected benefits for those not participating, and the average expected benefits, as outlined in equations (1), (2), (3). Furthermore, the replication dynamic equation for fishery enterprises engaging in the BBF construction strategy is computed as demonstrated in equation (4).

(1) $$u_{x}=z\left(r_{x}-p_{x}+a_{1}m\right)+\left(1-z\right)\left(r_{x}-p_{x}\right)=r_{x}-p_{x}+za_{1}m$$

(2) $$u_{1-x}=z\left(r_{1-x}-p_{1-x}\right)+\left(1-z\right)\left(r_{1-x}-p_{1-x}\right)=r_{1-x}-p_{1-x}$$

(3) $$\bar{u_{x}}=x\left(r_{x}-p_{x}+za_{1}m\right)+\left(1-x\right)\left(r_{1-x}-p_{1-x}\right)$$

(4) $$F\left(x\right)=x\left(u_{x}-\bar{u_{x}}\right)=x\left(1-x\right)\left(r_{x}-p_{x}+za_{1}m-r_{1-x}+p_{1-x}\right)$$

According to the evolutionary game benefit matrix of BBF construction in Table 1, we get the expected benefit of the fishermen supporting the BBF construction can be obtained in equation (5), the expected benefit of the fishermen not supporting the BBF construction in equation (6), and the average expected benefit in equation (7). Thus, equation (8) represents the replication dynamic equation for the strategy of supporting BBF construction among fishermen.

(5) $$u_{y}=z\left(r_{y}-p_{y}+a_{2}m\right)+\left(1-z\right)\left(r_{y}-p_{y}\right)=r_{y}-p_{y}+za_{2}m$$

(6) $$u_{1-y}=z\left(r_{1-y}-p_{1-y}\right)+\left(1-z\right)\left(r_{1-y}-p_{1-y}\right)=r_{1-y}-p_{1-y}$$

(7) $$\bar{u_{y}}=y\left(r_{y}-p_{y}+za_{2}m\right)+\left(1-y\right)\left(r_{1-y}-p_{1-y}\right)$$

(8) $$F\left(y\right)=y\left(u_{y}-\bar{u_{y}}\right)=y\left(1-y\right)\left(r_{y}-p_{y}+za_{2}m-r_{1-y}+p_{1-y}\right)$$

Referring to the evolutionary game benefit matrix for BBF construction in Table 1, we determine the government's expected benefit from subsidizing BBF construction, the expected benefit of not subsidizing BBF construction, and the average expected benefit, as indicated in equations (9), (10), (11). The replication dynamic equation of the government's BBF construction subsidies strategy is obtained in equation (12).

(9) $$u_{z}=r_{z}-xa_{1}m-ya_{2}m-p_{z}$$

(10) $$u_{1-z}=r_{1-z}-p_{1-z}$$

(11) $$\bar{u_{z}}=z\left(r_{z}-xa_{1}m-ya_{2}m-p_{z}\right)+\left(1-z\right)\left(r_{1-z}-p_{1-z}\right)$$

(12) $$F\left(z\right)=z\left(u_{z}-\bar{u_{z}}\right)=z\left(1-z\right)\left(r_{z}-p_{z}-xa_{1}m-ya_{2}m-r_{1-z}+p_{1-z}\right)$$

#### 3.2.2 The construction of evolutionary game system

The replicated dynamic system of the tripartite game involving government, fishermen and fishery enterprises is obtained based on the previous replicated dynamic equations (4), (8), (12). The Jacobi matrix $J_{3\times 3}$ is shown in equation (13).

(13) $$\begin{array}{c} J_{3\times 3}=\left(\begin{array}{cc} \begin{array}{cc} J_{11} & J_{12} \end{array} & J_{13}\\ \begin{array}{cc} \begin{array}{c} J_{21}\\ J_{31} \end{array} & \begin{array}{c} J_{22}\\ J_{32} \end{array} \end{array} & \begin{array}{c} J_{23}\\ J_{33} \end{array} \end{array}\right)=\left(\begin{array}{cc} \begin{array}{cc} \frac{\partial F\left(x\right)}{\partial x} & \frac{\partial F\left(x\right)}{\partial x} \end{array} & \frac{\partial F\left(x\right)}{\partial z}\\ \begin{array}{cc} \begin{array}{c} \frac{\partial F\left(y\right)}{\partial x}\\ \frac{\partial F\left(z\right)}{\partial x} \end{array} & \begin{array}{c} \frac{\partial F\left(y\right)}{\partial x}\\ \frac{\partial F\left(z\right)}{\partial x} \end{array} \end{array} & \begin{array}{c} \frac{\partial F\left(y\right)}{\partial z}\\ \frac{\partial F\left(z\right)}{\partial z} \end{array} \end{array}\right) \end{array}$$

The multi-subject evolutionary game's stable solutions are necessarily pure strategy Nash equilibria. So, we put 8 pure strategy equilibrium points into the Jacobi matrix $J_{3\times 3}$, then we get three eigenvalues $k_{1}$, $k_{2}$ and $k_{3}$. In summary, the eigenvalues of equilibrium points are shown in Table 2.

**Table 2:** Eigenvalues of equilibrium points.

	Eigenvalue $k_{1}$	Eigenvalue $k_{2}$	Eigenvalue $k_{3}$
E_1_(0,0,0)	$r_{x}-p_{x}-r_{1-x}+p_{1-x}$	$r_{y}-p_{y}-r_{1-y}+p_{1-y}$	$r_{z}-r_{1-z}$
E_2_(1,0,0)	$-\left(r_{x}-p_{x}-r_{1-x}+p_{1-x}\right)$	$-\left(r_{y}-p_{y}-r_{1-y}+p_{1-y}\right)$	$-\left(r_{z}-r_{1-z}-a_{1}m\right)$
E_3_(0,1,0)	$r_{x}-p_{x}-r_{1-x}+p_{1-x}$	$-\left(r_{y}-p_{y}-r_{1-y}+p_{1-y}\right)$	$r_{z}-r_{1-z}-a_{2}m$
E_4_(0,0,1)	$r_{x}-p_{x}-r_{1-x}+p_{1-x}+a_{1}m$	$r_{y}-p_{y}-r_{1-y}+p_{1-y}+a_{2}m$	$-\left(r_{z}-r_{1-z}\right)$
E_5_(1,1,0)	$-\left(r_{x}-p_{x}-r_{1-x}+p_{1-x}\right)$	$-\left(r_{y}-p_{y}-r_{1-y}+p_{1-y}\right)$	$r_{z}-r_{1-z}-m$
E_6_(1,0,1)	$-\left(r_{x}-p_{x}-r_{1-x}+p_{1-x}+a_{1}m\right)$	$r_{y}-p_{y}-r_{1-y}+p_{1-y}+a_{2}m$	$-\left(r_{z}-r_{1-z}-a_{1}m\right)$
E_7_(0,1,1)	$r_{x}-p_{x}-r_{1-x}+p_{1-x}+a_{1}m$	$-\left(r_{y}-p_{y}-r_{1-y}+p_{1-y}+a_{2}m\right)$	$-\left(r_{z}-r_{1-z}-a_{2}m\right)$
E_8_(1,1,1)	$-\left(r_{x}-p_{x}-r_{1-x}+p_{1-x}+a_{1}m\right)$	$-\left(r_{y}-p_{y}-r_{1-y}+p_{1-y}+a_{2}m\right)$	$-\left(r_{z}-r_{1-z}-m\right)$

From the system stability principle, it is known that the eight pure strategy equilibrium points have stability if three eigenvalues are negative. The stability of equilibrium points can be assessed by examining the eigenvalues of the Jacobi matrix, as detailed in Table 2. Additionally, Table 3 outlines the three stability conditions for each equilibrium point of the replicated dynamic system.

**Table 3:** Stability conditions of eight pure strategy equilibrium points.

	Condition 1	Condition 2	Condition 3
E_1_(0,0,0)	$r_{x}-p_{x}<r_{1-x}-p_{1-x}$	$r_{y}-p_{y}<r_{1-y}-p_{1-y}$	$r_{z}<r_{1-z}$
E_2_(1,0,0)	$r_{x}-p_{x}>r_{1-x}-p_{1-x}$	$r_{y}-p_{y}>r_{1-y}-p_{1-y}$	$r_{z}-a_{1}m>r_{1-z}$
E_3_(0,1,0)	$r_{x}-p_{x}<r_{1-x}-p_{1-x}$	$r_{y}-p_{y}>r_{1-y}-p_{1-y}$	$r_{z}-a_{2}m<r_{1-z}$
E_4_(0,0,1)	$r_{x}-p_{x}+a_{1}m<r_{1-x}-p_{1-x}$	$r_{y}-p_{y}+a_{2}m<r_{1-y}-p_{1-y}$	$r_{z}>r_{1-z}$
E_5_(1,1,0)	$r_{x}-p_{x}>r_{1-x}-p_{1-x}$	$r_{y}-p_{y}>r_{1-y}-p_{1-y}$	$r_{z}-m<r_{1-z}$
E_6_(1,0,1)	$r_{x}-p_{x}+a_{1}m>r_{1-x}-p_{1-x}$	$r_{y}-p_{y}+a_{2}m<r_{1-y}-p_{1-y}$	$r_{z}-a_{1}m>r_{1-z}$
E_7_(0,1,1)	$r_{x}-p_{x}+a_{1}m<r_{1-x}-p_{1-x}$	$r_{y}-p_{y}+a_{2}m>r_{1-y}-p_{1-y}$	$r_{z}-a_{2}m>r_{1-z}$
E_8_(1,1,1)	$r_{x}-p_{x}+a_{1}m>r_{1-x}-p_{1-x}$	$r_{y}-p_{y}+a_{2}m>r_{1-y}-p_{1-y}$	$r_{z}-m>r_{1-z}$

### 3.3 Numerical simulation method

The behavioral choices of each subject are not constant, but influenced by the behavioral choices of other subjects. In order to simulate the influence of parameter changes on the game behavior of each subject in the BBF construction, this paper replicates the dynamic equation system based on the evolutionary game. In order to conduct a more in-depth analysis of the lasting effects of the subsidies amount for BBF construction, this paper sets the basic evolution time as 1000 and 2023 as the base period. From the social survey, we get the parameters of numerical simulation model in Table 4.

**Table 4:** Parameter assignment of the simulation model.

	Explanation	Assignment	Unit of measurement	Data acquisition
$r_{x}$	The benefits of enterprises participate in the BBF construction	0.5	Million per year	Survey of fishery enterprises
$p_{x}$	The cost of enterprises participates in the BBF construction	0.25	Hundred thousand per year	Survey of fishery enterprises
$r_{1-x}$	The benefits of fishery enterprises do not participate in the BBF construction	0.4	Million per year	Survey of fishery enterprises
$p_{1-x}$	The cost of fishery enterprises does not participate in the BBF construction	0.1	Hundred thousand per year	Survey of fishery enterprises
$r_{y}$	The benefits of fishermen support BBF construction	0.5	Hundred thousand per year	Survey of fishermen
$p_{y}$	The cost of fishermen supports BBF construction	0.1	Hundred thousand per year	Survey of fishermen
$r_{1-y}$	The benefits of fishermen do not support BBF construction	0.6	Hundred thousand per year	Survey of fishermen
$p_{1-y}$	The cost of fishermen does not support BBF construction	0.2	Hundred thousand per year	Survey of fishermen
$r_{z}$	The utility of government subsidizing BBF	0.9	None	Investigation of government
$p_{z}$	The cost of government subsidizing BBF	0.45	Million per year	Investigation of government
$r_{1-z}$	The utility of government not subsidizing BBF	0.5	None	Investigation of government
$p_{1-z}$	The cost of government not subsidizing BBF	0.3	Million per year	Investigation of government
m	Total government subsidies for BBF construction	0.2	One billion per year	Investigation of government
$a_{1}$	Subsidies coefficient for fishery enterprises	0.5	None	Investigation of government
$a_{2}$	Subsidies coefficient for fishermen	0.5	None	Investigation of government

From Table 4, we can know that the costs and benefits of fishery enterprises participating and not participating in the BBF construction are the fishery enterprises’ average values surveyed, the benefits of participation $r_{x}=0.5$, the costs of participation $p_{x}=0.25$, the subsidies coefficient of participation $a_{1}=0.5$, the benefits of non-participation $r_{1-x}=0.4$, and the costs of non-participation $p_{1-x}=0.1$. The costs and benefits of fishermen who support and do not support the BBF construction are the average values of the surveyed fishermen, the benefits of support $r_{y}=0.5$, the costs of support $p_{y}=0.1$, the subsidies coefficient of support $a_{2}=0.5$, the benefits of non-support $r_{1-y}=0.6$, the costs of non-support $p_{1-y}=0.2$. The amount of fishery subsidies for the BBF construction m=0.2, the utility gained $r_{z}=0.9$ and the associated costs $p_{z}=0.45$. The utility of not subsidizing the BBF construction by the government $r_{1-z}=0.5$ and the associated costs $p_{1-z}=0.3$.

## 4 Results and discussion

### 4.1 Evolutionary game model results

#### 4.1.1 Stabilization strategy for fishery enterprises to participate in the BBF construction

According to equation (4), this paper set $f\left(z\right)=r_{x}-p_{x}+za_{1}m-r_{1-x}+p_{1-x}$, since $\partial f\left(z\right)/\partial z=a_{1}m\geq 0$, it is clear that f(z) is monotone increasing. Then we can get F(x)=x(1−x)f(z) and its' first order derivative $F^{\prime }\left(x\right)=\left(1-2x\right)f\left(z\right)$ from equation (4). Set f(z)=0, and calculate $z_{0}=-\left(r_{x}-p_{x}-r_{1-x}+p_{1-x}\right)/a_{1}m$. To maintain the stability of the fishery enterprise's strategy, it is necessary to fulfill the requirements of F(x)=0 and $F^{\prime }\left(x\right)<0$. Combining the above analysis, we can infer that if $z=z_{0}$, x∈[0,1] is stable, and the fishery enterprise cannot achieve a stabilization strategy in such a situation. If $z<z_{0}$, x=0 is stable. In this case, fishery enterprise will not participate in BBF construction. When $z>z_{0}$, x=1 is stable. In this case, fishery enterprise will participate in the BBF construction.

Combining the above analysis, it is clear that if subsidies' probability is low, fishery enterprises tend not to participate in the BBF construction, and when government subsidies’ probability is high, fishery enterprises tend to participate in the BBF construction. This indicates that fishery subsidies have a positive influence on fishery enterprises' participation in the BBF construction. It can be seen that the provision of fishery subsidies contributed to the BBF construction.

#### 4.1.2 Stabilization strategy for fishermen to support the BBF construction

According to equation (8), this paper set $f\left(z\right)=r_{y}-p_{y}+za_{2}m-r_{1-y}+p_{1-y}$, since $\partial f\left(z\right)/\partial z=za_{2}m\geq 0$, so f(z) is a monotone increasing function with respect to z. Then we can get F(y)=y(1−y)f(z) from equation (8), and $F^{\prime }\left(y\right)=\left(1-2y\right)f\left(z\right)$ is the first order derivative. Set f(z)=0, and calculate $z_{0}=-\left(r_{y}-p_{y}-r_{1-y}+p_{1-y}\right)/a_{2}m$. To enable fishermen to achieve a stable strategy, it is necessary to achieve F(y)=0 and $F^{\prime }\left(y\right)<0$. Combining above analysis, we infer that if $z=z_{0}$, y∈[0,1] is stable. In such a situation, fishermen's stable strategy is uncertain. When $z<z_{0}$, y=0 is stable. In such a situation, fishermen's stable strategy is not to support the BBF construction. When $z>z_{0}$, y=1 is stable. In such a situation, fishermen employ a stabilization strategy by endorsing the construction of the BBF.

Combining the above analysis, it is clear that if government subsidies' probability is low, fishermen choose not to support the BBF construction, and if government subsidies' probability is high, fishermen choose to support the BBF construction. This suggests that fishery subsidies have a beneficial impact on fishermen's behavior of supporting the BBF construction.

#### 4.1.3 Stabilization strategy of government subsidized BBF construction

According to equation (12), this paper set $f\left(x\right)=r_{z}-p_{z}-xa_{1}m-ya_{2}m-r_{1-z}+p_{1-z}$, since $\partial f\left(x\right)/\partial x=-a_{1}m\leq 0$, f(x) is monotone decreasing. Then we can get F(z)=z(1−z)f(x) and its' first order derivative $F^{\prime }\left(z\right)=\left(1-2z\right)f\left(x\right)$ from equation (12). Set f(x)=0, and calculate $x_{0}=\left(r_{z}-p_{z}-ya_{2}m-r_{1-z}+p_{1-z}\right)/a_{1}m$. To enable government to achieve a stable strategy, it is necessary to achieve F(z)=0 and $F^{\prime }\left(z\right)<0$. Combining the above analysis, we can infer that if $x=x_{0}$, z∈[0,1] is stable. In such a situation, the government's stable strategy is uncertain. When $x<x_{0}$, z=1 is stable. In such a situation, government's stabilization strategy involves offering subsidies for BBF construction. When $x>x_{0}$, z=0 is stable, and the government's stabilization strategy involves not providing subsidies for BBF construction.

The aforementioned findings reveal that in scenarios where the likelihood of fishery enterprises participating in BBF construction is low, the government leans towards subsidizing the BBF construction. Conversely, when the probability of fishery enterprises participating in BBF construction is high, the government opts not to provide subsidies for the BBF construction.

According to equation (12), this paper set $f\left(y\right)=r_{z}-p_{z}-xa_{1}m-ya_{2}m-r_{1-z}+p_{1-z}$, since $\partial f\left(y\right)/\partial y=-a_{2}m\leq 0$, f(y) is monotone decreasing. Then we can get F(z)=z(1−z)f(y) and its' first order derivative $F^{\prime }\left(z\right)=\left(1-2z\right)f\left(y\right)$ from equation (12). Set f(y)=0, and calculate $y_{0}=\left(r_{z}-p_{z}-xa_{1}m-r_{1-z}+p_{1-z}\right)/a_{2}m$. To maintain the stability of the fishery subsidies strategy, it is necessary to fulfill the requirements of F(z)=0 and $F^{\prime }\left(z\right)<0$. Combining the above analysis, we can infer that while $y=y_{0}$, z∈[0,1] is stable. In such a situation, government's stable strategy is uncertain. If $y<y_{0}$, z=1 is stable. In this situation, the government's stabilization strategy involves subsidizing the BBF construction. When $y>y_{0}$, z=0 is stable. In such a situation, the government's stabilization strategy involves refraining from subsidizing BBF construction.

Combining the above analysis, the government subsidizes BBF construction if the probability of fishermen supporting BBF construction is low. While the government does not subsidize BBF construction if fishermen's probability of supporting BBF construction is high.

#### 4.1.4 Evolutionary game system's stability analysis of BBF construction

First, in Table 3, when $r_{x}-p_{x}<r_{1-x}-p_{1-x}$, $r_{y}-p_{y}<r_{1-y}-p_{1-y}$, $r_{z}<p_{1-z}$, E_1_(0,0,0) is stable. It is shown that if the profit of fishery enterprises participating in the BBF construction is less than the profit of not participating in the BBF construction, the profit of fishermen supporting the BBF construction is less than the profit of not supporting the BBF construction, and the effectiveness of government subsidies does not exceed the effectiveness not subsidizing, the pure strategy equilibrium point is stable. In such a situation, fishery enterprises do not participate in the BBF construction and the fishermen do not support the BBF construction. The government does not subsidize the BBF construction.

Second, when $r_{x}-p_{x}>r_{1-x}-p_{1-x}$, $r_{y}-p_{y}>r_{1-y}-p_{1-y}$, $r_{z}-a_{1}m>r_{1-z}$, E_2_(1,0,0) is stable. That is, when the profit of fishery enterprises participating in the BBF construction exceeds the profit of not participating in the BBF construction, the profit of fishermen supporting the BBF construction is higher than not supporting the BBF construction, and effectiveness of government subsidies minus the fishery enterprises subsidies exceeds effectiveness of no subsidies, the pure strategy equilibrium point is stable. In such a situation, fishery enterprises participate in the BBF construction, fishermen do not support the BBF construction, and the government does not subsidize the BBF construction.

Third, when $r_{x}-p_{x}<r_{1-x}-p_{1-x}$, $r_{y}-p_{y}>r_{1-y}-p_{1-y}$, $r_{z}-a_{2}m<r_{1-z}$, E_3_(0,1,0) is stable. That is, when the profit of fishery enterprises participating in the BBF construction is less than the profit of not participating in the BBF construction, the profit of fishermen supporting the BBF construction is higher than the profit of not supporting the BBF construction, and effectiveness of government subsidies minus fishermen subsidies does not exceed effectiveness of not subsidizing, the pure strategy equilibrium point is stable. In such a situation, fishery enterprises do not participate in the BBF construction, fishermen support the BBF construction and the government does not subsidize BBF construction.

Fourth, when $r_{x}-p_{x}+a_{1}m<r_{1-x}-p_{1-x}$, $r_{y}-p_{y}+a_{2}m<r_{1-y}-p_{1-y}$, $r_{z}>p_{1-z}$, E_4_(0,0,1) is stable. The analysis reveals that if the combined profit and subsidies for fishery enterprises engaging in BBF construction is lower than the profit of abstaining from BBF construction, and if the sum of profit and subsidies for fishermen supporting BBF construction does not surpass the profit of fishermen supporting BBF construction, then the efficacy of government subsidies surpasses that of not providing subsidies, rendering the pure strategy equilibrium point stable. In such a situation, fishery enterprises do not participate in the BBF construction, fishermen do not support the BBF construction, and the government subsidizes BBF construction.

Fifth, when $r_{x}-p_{x}>r_{1-x}-p_{1-x}$, $r_{y}-p_{y}>r_{1-y}-p_{1-y}$, $r_{z}-m<r_{1-z}$, E_5_(1,1,0) is stable. In other words, if the profit generated by fishery enterprises participating in BBF construction surpasses the profit of refraining from BBF construction, the profit derived from fishermen supporting BBF construction is greater than the profit of not supporting it, and the effectiveness of government subsidies minus the cost of fishery subsidies does not outstrip the effectiveness of no subsidies, then the pure strategy equilibrium point is stable. In such a situation, fishery enterprises participate in the BBF construction, fishermen support the BBF construction, and the government does not subsidize BBF construction.

Sixth, when $r_{x}-p_{x}+a_{1}m>r_{1-x}-p_{1-x}$, $r_{y}-p_{y}+a_{2}m<r_{1-y}-p_{1-y}$, $r_{z}-a_{1}m>r_{1-z}$, E_6_(1,0,1) is stable. In other words, if the combined profits of fishery enterprises participating in BBF construction, along with the received subsidies, surpass the profits of those abstaining from BBF construction, and if the combined profits of fishermen supporting BBF construction, along with the received subsidies, exceed the profits of those not participating in BBF construction, while the effectiveness of government subsidies minus the utility of fishery fishermen subsidies surpasses the effectiveness of no subsidies, then the pure strategy equilibrium point is deemed stable. In such a situation, fishery enterprises participate in the BBF construction, fishermen do not support the BBF construction, and the government subsidizes BBF construction.

Seventh, when $r_{x}-p_{x}+a_{1}m<r_{1-x}-p_{1-x}$, $r_{y}-p_{y}+a_{2}m>r_{1-y}-p_{1-y}$, $r_{z}-a_{2}m>r_{1-z}$, E_7_(0,1,1) is stable. That is, if profits of fishing enterprises participating in BBF construction combined with the amount of subsidies received are less than the profits of those not participating in BBF construction, the profits of fishermen supporting BBF construction combined with the amount of subsidies received are less than the profits of those not participating in BBF construction, effectiveness of government subsidies minus fishermen subsidies exceed effectiveness of no subsidies, the pure strategy equilibrium point is stable. In such a situation, fishery enterprises do not participate in the BBF construction, fishermen support the BBF construction, and the government subsidizes BBF construction.

Eighth, when $r_{x}-p_{x}+a_{1}m>r_{1-x}-p_{1-x}$, $r_{y}-p_{y}+a_{2}m>r_{1-y}-p_{1-y}$, $r_{z}-m>r_{1-z}$, E_8_(1,1,1) is stable. It is show that if the profits of fishing firms participating in the BBF construction combined with the amount of subsidies obtained are higher than the profits of those not participating in the BBF construction, the profits of fishermen supporting the BBF construction combined with the amount of subsidies obtained are higher than the profits of those not participating in the BBF construction, and effectiveness of government subsidies minus all subsidies amount exceed effectiveness of no subsidies, the pure strategy equilibrium point is stable. In such a situation, fishery enterprises participate in the BBF construction, the fishermen support the BBF construction, and the government subsidizes BBF construction.

### 4.2 Numerical simulation

#### 4.2.1 Impact of subsidies amount for the BBF construction

**Fig. 3:**
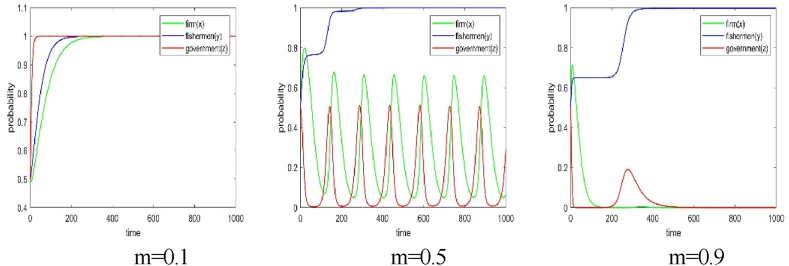
The effect of total government subsidies on each subject's game strategy.

**Fig. 4:**
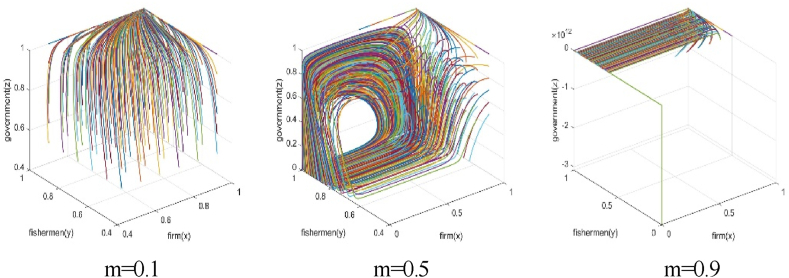
Effect of total government subsidies on the system's stability.

The numerical simulation employs the replicated dynamic equation system mentioned above as the simulation model, with parameters assigned as per Table 4. The visualization of simulation results is conducted using the Plot function in Matlab2019b software. Additionally, the simulation results can be displayed in three-dimension using the Plot3 function. To simulate the effects of subsidies amount on the behavior strategy of each subject and the game system's stability, the numerical simulation results of m=0.1, m=0.5, and m=0.9 are presented in Fig. 3, Fig. 4.

It is evident that when government subsidies amount for the BBF construction is 0.1, fishery enterprises participate in the BBF construction, fishermen support the BBF construction, and the government subsidizes the BBF construction. In such a situation, the three parties achieve a stable behavior strategy at (1,1,1). As the total amount of fishery subsidies for the BBF construction increases to 0.5, the fishermen support the BBF construction, but the strategies of the fishery enterprises and the government fluctuate. In such a situation, the three parties’ strategy are not stable. When the fishery subsidies for the BBF construction are further increased to 0.9, fishermen support the BBF construction, but fishery enterprises do not participate in the BBF construction. In such a situation, the three parties achieve a stable behavior strategy at (0,1,0). As the fishery subsidies increases, the fishery enterprises gradually do not participate in the BBF construction, and the government gradually removes the subsidies when the burden of subsidies increases.

#### 4.2.2 Impact of different subsidies subject for the BBF construction

**Fig. 5:**
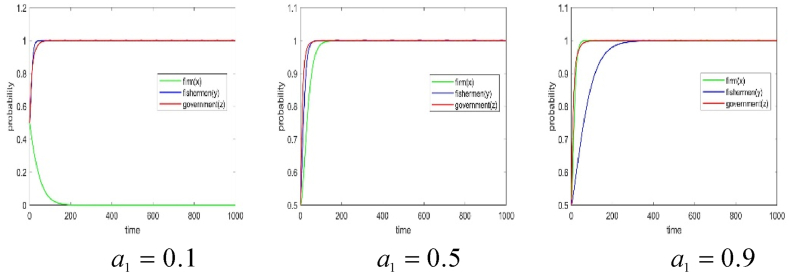
Effect of subsidies for fishery enterprises on the game strategies of the parties.

**Fig. 6:**
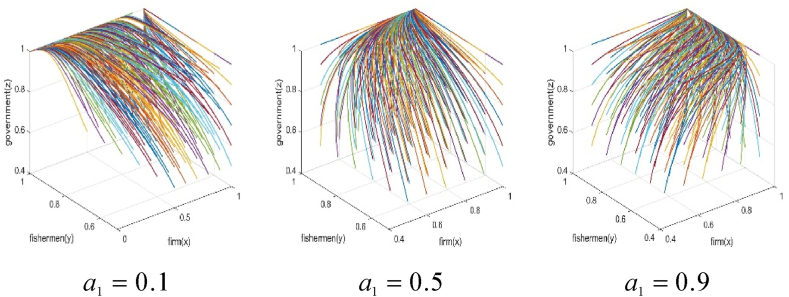
Effect of subsidies for fishery enterprises on the game system's stability.

(1) The impact of subsidies for fishery enterprises. In order to simulate the influence of the change of subsidies on the BBF construction by fishery enterprises on the behavior strategy of each subject, the numerical simulation results of $a_{1}=0.1$, $a_{1}=0.5$, $a_{1}=0.9$ are presented in Fig. 5, Fig. 6.

It is evident that when the subsidies coefficient for BBF construction in fishery enterprises is 0.1, the fishermen supports the BBF construction, and the government subsidizes it, but the fishery enterprises do not participate. In such a situation, the three parties achieve a stable behavioral strategy at (0, 1, 1). When the subsidies coefficients for BBF construction in fishery enterprises are 0.5 and 0.9, fishery enterprises participate in the BBF construction, fishermen support it, and the government subsidizes it. In such a situation, the three parties achieve a stable behavioral strategy at (1, 1, 1). This indicates that subsidies for BBF construction in fishery enterprises generate motivational effects. As subsidies for BBF construction increase, fishery enterprises gradually choose to participate in it. This may be attributed to the fact that when government subsidies are too low, fishery enterprises do not benefit from participating in BBF construction because the benefits are much lower than the costs and inputs they pay.

**Fig. 7:**
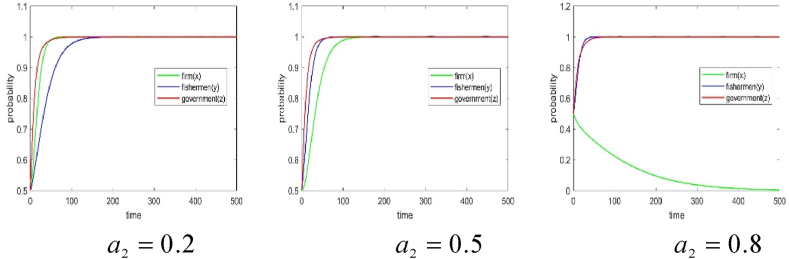
Effect of fishermen's subsidies.

**Fig. 8:**
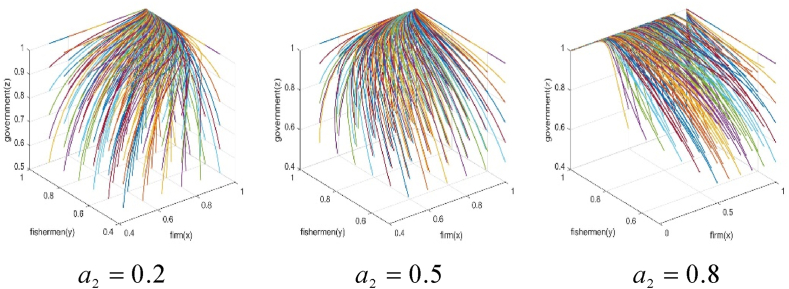
Effect of fishermen's subsidies on the game system's stability.

(2) The impact of fishermen subsidies. We set $a_{2}=0.2$, $a_{2}=0.5$, $a_{2}=0.8$ respectively, then simulate the influence of subsidies change for the BBF construction by fishermen on the behavior strategy of each subject and the system stability. The results of our numerical simulations are presented in Fig. 7, Fig. 8.

It is evident that when the subsidies coefficients for fishermen supporting the BBF construction are 0.2 and 0.5, fishery enterprises participate in the BBF construction, fishermen support the BBF construction, and the government subsidizes the BBF construction. In such a situation, the three parties achieve a stable behavior strategy at (1,1,1). However, when the subsidies coefficient for fishermen supporting the BBF construction is 0.8, fishermen support the BBF construction and the government subsidizes the BBF, but fishery enterprises gradually do not participate in the BBF construction. In such a situation, the three parties achieve a stable behavior strategy at (0,1,1). The above results show that subsidies for fishermen generate motivational effects. As subsidies for fishermen increase, fishermen actively withdraw from offshore fishing to support the BBF construction, but fishery enterprises gradually do not participate in the BBF construction. This phenomenon could be attributed to the finite overall allocation of government subsidies, where the escalation in subsidies for fishermen has displaced the available funds allocated for fishery enterprises.

In summary, although the withdrawal of fishermen from fishing is a major livelihood issue, increasing subsidies for their withdrawal is particularly important in the BBF construction. Nevertheless, in the scenario where the total amount of government subsidies remains constant, it becomes essential to judiciously allocate subsidies for BBF construction to fishery enterprises. This strategic allocation aims to expedite the attainment of BBF construction goals. Due to financial and technical constraints, a large area of the ocean has not been developed yet. If only the BBF ocean area is established without fishery enterprises to develop farming, the establishment of BBF will lose its meaning. This may also be the reason why most countries have not established BBF until now.

### 4.3 Discussion

This study is concerned with government's subsidies strategy in the BBF construction, which includes four sub-questions: whether to subsidize, when to subsidize, how much to subsidize, and whom to subsidize. The findings show that government subsidies can increase the probability of fishery enterprises participating in BBF construction and fishermen supporting it. However, the government's subsidies strategy varies across eight different situations. For subsidies object, the government subsidies should not be too high. In terms of subsidies amount, subsidizing fishery enterprises is more effective than subsidizing fishermen. The results of these studies answer the above four questions.

The effects of fishery subsidies have been thoroughly examined in studies. Owusu and Adjei identified that about 95% of China's fishery subsidies have a detrimental impact on the sustainable development of fisheries [41]. Dağtekin et al. pointed out that fuel subsidies have led to over-capitalization of fisheries in Turkey and Georgia and advocate the elimination of fuel subsidies for fisheries [46]. On the contrary, this paper verifies the enthusiasm of subsidies and argues that fishery subsidies should be provided. Chen et al. analyzed the regulatory and subsidies mechanisms of China's farmland protection policy and find that regulatory and subsidies instruments have alternative relationships [26]. Since the BBF construction has significant ecological and economic effects, it does not belong to the scope of environmental regulation, so this paper only considers the subsidies mechanism. Zhang et al. found that perceived financial value, subsidies, and advocacy are essential for farmers' willingness to participate in farmland protection cooperation [24]. In line with this discovery, this paper similarly explores the government's role in farmland conservation and examines the impact of government subsidies on BBF construction. Wan et al. indicated that subsidies can motivate marine ranch enterprises to improve their carbon sink capacity, but the game system does not present a stable evolutionary strategy when the subsidies are too high [47]. The conclusion is consistent with this paper, that the BBF construction should adhere to the principle of moderate subsidies. Zheng and Yu found that subsidizing carbon sink fishery producers is more effective than subsidizing consumers in achieving government subsidies policy goals [39]. This paper makes a similar finding, arguing that subsidizing fishery enterprises is more conducive to the BBF construction. Mallory found that inequities in the distribution of fishery subsidies affect the livelihoods of vulnerable groups of small-scale fishermen [44]. It is a warning that countries should allocate subsidies for the construction of “Blue Basic Farmland” fairly and reasonably. Qian et al. comprehensively evaluate the soil quality and site conditions of farmland to achieve scientific zoning for the protection of basic farmland [20]. This conclusion provides a basis for the quality division of aquaculture sea areas and the classification and protection of BBF. Fiedler et al. conducted a cost-benefit evaluation of a nationwide household pool mola fish extension project in Bangladesh [48]. It is also a reminder of the need for a cost-benefit analysis of Blue Prime Farmland. Lu et al. pointed out that arable land conservation policies should be based on the perspective of enhancing farmers' interests [22]. This is especially true for the BBF construction, and the resettlement of fishermen who have retired from fishing should be properly resolved.

The shortcomings of this paper are as follows. Chen et al. employed both the exponential approach and the food requirement approach to predict the scale of basic farmland protection. They also determined the threshold value of the basic farmland scale by evaluating the forecasted results [5]. This paper only proposes the idea of building BBF without further calculating the amount of BBF based on food demand. In addition, the BBF construction is a systematic project, involving many subjects and economic sectors, besides the fishery enterprises, fishermen and fishery management departments, which are closely related to marine fisheries, it may also involve marine management departments, maritime departments, marine environment management departments and so on. This paper only analyzes the importance of building BBF for national food security from the perspective of fisheries economy, without considering other economic sectors or non-economic factors.

## 5 Conclusions and policy implications

### 5.1 Conclusions

This paper presents a comprehensive analysis by constructing a three-party evolutionary game model involving the government, fishermen, and fishery enterprises. The subsequent examination of the government's subsidies strategy for BBF construction is supported by a simulation analysis based on survey data. The key findings indicate that government subsidies play a pivotal role in promoting BBF construction, positively influencing the participation of fishery enterprises and garnering support from fishermen. Furthermore, the dynamic nature of the game system leads to eight distinct steady states, prompting adjustments in the optimal government subsidies strategy. It is recommended to maintain fishery subsidies within reasonable limits, as an excessive amount negatively impacts system stability. Notably, subsidies for fishery enterprises are more effective than those for fishermen in promoting BBF construction, positively impacting system stabilization, while subsidies to fishermen have a destabilizing effect on the evolutionary game system.

### 5.2 Policy implications

Policy implications arising from the study suggest a multifaceted approach. Firstly, the government ought to establish subsidies dedicated to BBF construction. This entails setting up subsidies for artificial reef construction and the acquisition of deep-water nets, aiming to alleviate the financial burden on fishery enterprises engaging in BBF construction. Simultaneously, subsidies for seawater shellfish farming, excluding feeding costs and drawing inspiration from food production subsidies, should be implemented. Secondly, it is crucial for the government to judiciously manage the subsidies amounts. In the initial phase of BBF construction, a moderate increase in subsidies is recommended, with equal subsidies distributed to both fishery enterprises and fishermen. As BBF construction progresses, the government can gradually decrease the subsidies amounts. Upon the establishment of the basic blue farmland, subsidies can be phased out. Thirdly, the focal point of subsidies should be directed towards fishery enterprises. This targeted approach serves to alleviate the ocean use fees for enterprises participating in BBF construction, subsequently reducing development costs and facilitating the transition of marine food development from near-shore to offshore waters.

## Ethical approval

The submitted manuscript is original and have not been published elsewhere in any form or language.

## Consent to participate

Done.

## Consent to publish

All authors agreed with the content and that all gave explicit consent to submit.

## Data availability

Data included in article/supp. material/referenced in article.

## Funding

Not available.

## Statement on data availability

Data included in article.

## CRediT authorship contribution statement

**Zicheng Wang:** Writing – original draft, Methodology, Investigation, Funding acquisition, Formal analysis, Data curation, Conceptualization. **Shan Zheng:** Supervision, Software, Resources, Project administration, Methodology.

## Declaration of competing interest

The authors declare that they have no known competing financial interests or personal relationships that could have appeared to influence the work reported in this paper.
